# Supplementary material for: Organisational and neuromodulatory underpinnings of structural-functional connectivity decoupling in patients with Parkinson’s disease
Source: Commun Biol. 2021 Jan 19;4:86. doi: 10.1038/s42003-020-01622-9 (PMC7815846; doi:10.1038/s42003-020-01622-9)
Supplement: Supplementary file 2 — Supplementary Information [file 42003_2020_1622_MOESM2_ESM.pdf]

SUPPLEMENTARY MATERIAL

Organisational and neuromodulatory underpinnings of structural-functional connectivity decoupling in patients with Parkinson's disease

Angeliki Zarkali[1], Peter McColgan[2], Louise-Ann Leyland[1], Andrew J. Lees[3], Geraint Rees[4,5], Rimona S. Weil[1,5,6]

Contents

Supplementary Figure 1 2
Supplementary Figure 2 3
Supplementary Table 1 4
Supplementary Table 2 5
Supplementary Figure 3 6
Supplementary Figure 4 7
Supplementary Figure 5 8
Supplementary Figure 6 9
Supplementary Table 3 10
URLs 11
References: 11

Supplementary Figure 1

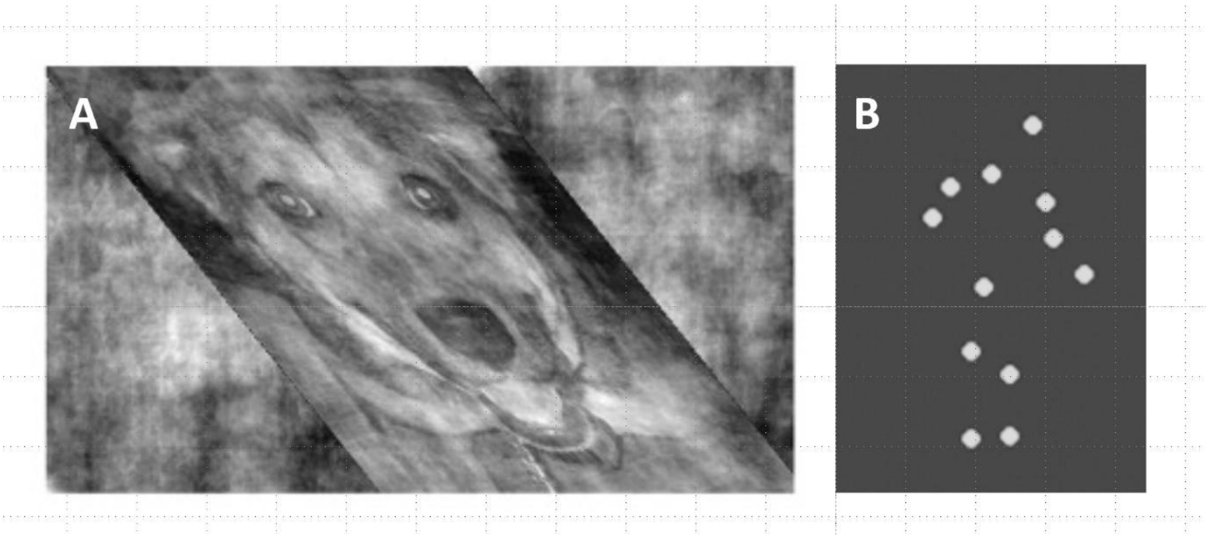

Examples of the computer-based visual stimuli
A: Cats and Dogs task: Example stimulus. Presentation time = 280 ms, with increasing visual skew applied. The task was to identify whether the image showed a dog or a cat. (90 repetitions). Details on stimulus production have been previously described[1]
B: Biological Motion task: Example frame from an animation depicting a person walking. Increasing number of moving dots were added. The task was to determine whether the moving stimulus showed a person or scrambled noise (225 repetitions).

Supplementary Figure 2

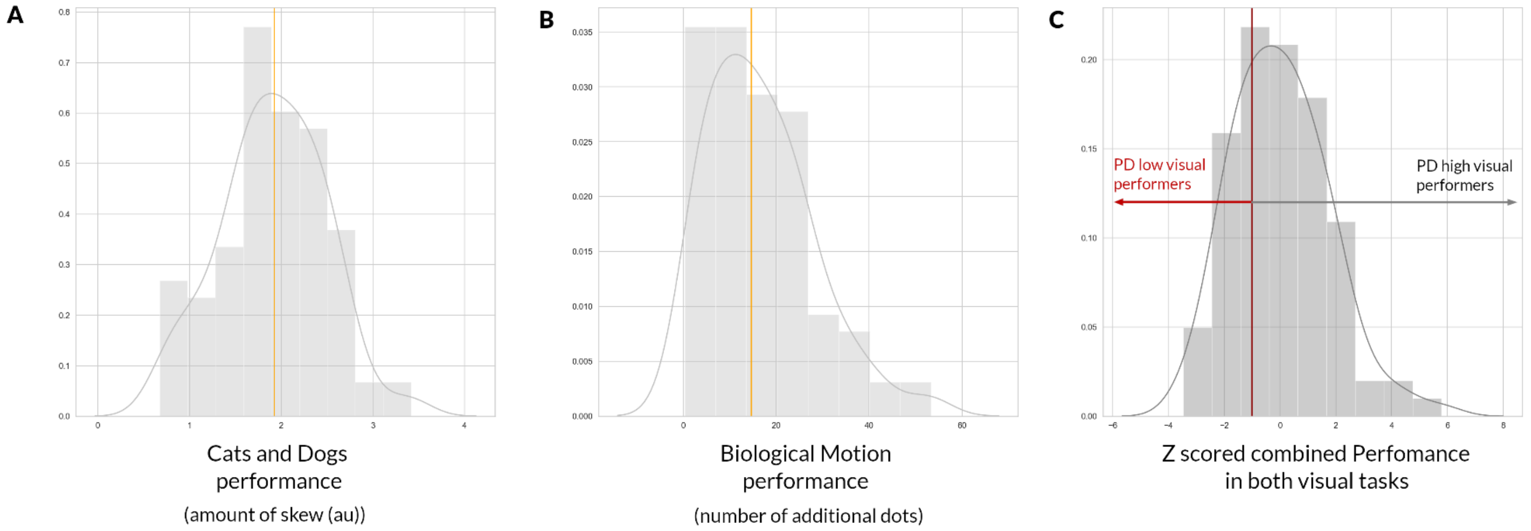

Performance of patients with Parkinson's disease in the visual tasks.
A. Distribution of performance in the Cats and Dogs task. Orange line highlights the median performance in this task. Performance was not significantly different between PD as a whole and controls (rho=0.168, p=0.057).
B. Distribution of performance in the Biological motion task. Orange line highlights the median performance in this task. Performance was not significantly different between PD as a whole and controls (rho=0.025, p=0.778).
C. Distribution of the combined Z score of performance in the Biological motion and Cats and Dogs task
($Z_{score} = Z_{Biolmotion} + Z_{cats\&dogs}$). The distribution on the left of the red line represents the patients who performed worse than the median (left of the orange lines in A and B) for both the Cats and Dogs and the Biological motion task. These were classified as PD low visual performers.

Supplementary Table 1

Supplementary Table 1. Detailed neuropsychological performance

Domain	Test	Control n=33	PD high visual performers n=58	PD low visual performers n=30	Statistic
**General cognition**	MOCA	**28.8 (1.3)**	**28.8 (1.3)**	**27.9 (2.4)**	**0.003[a,b]**
	MMSE	29 (1.1)	29 (1.1)	28.9 (1.3)	0.573
**Attention**	Digit span forwards	9.6 (1.7)	9.5 (2.1)	8.8 (2.2)	0.307
	Digit span backwards	7.4 (2.3)	7.2 (2.0)	6.8 (2.1)	0.306
	Stroop: Colour (sec)	32.9 (6.8)	32.2 (6.6)	37.2 (9.4)	0.082
**Executive function**	Stroop: Interference (sec)	**59.1 (16.9)**	**54.9 (11.5)**	**69.1 (23.9)**	**0.015 [a,b]**
	Category fluency	21.9 (5.6)	22.5 (5.2)	19.8 (6.9)	0.175
**Memory**	Word Recognition Task	**24.4 (0.9)**	**24.3 (1.2)**	**23.7 (1.3)**	**0.023[b]**
	Logical Memory (delayed)	13.7 (3.8)	14.1 (3.9)	12.3 (5.1)	0.422
**Language**	Graded Naming Task	24.1 (2.5)	23.5 (5.0)	23.2 (3.5)	0.409
	Letter Fluency	16.8 (4.6)	16.8 (5.2)	17.2 (6.8)	0.953
**Visuospatial**	Visual Object and Space Perception Battery	**56.3 (2.0)**	**54.1 (7.0)**	**53.0 (7.2)**	**0.002[b]**
	Hooper	**25.4 (2.9)**	**25.7 (2.1)**	**22.7 (2.7)**	**<0.001[a,b]**

All results presented as mean (standard deviation).

[a] statistically significant difference between PD high visual performers and PD low visual performers

[b] statistically significant difference between PD low visual performers and controls

Supplementary Table 2

Supplementary Table 2. List of selected genes encoding neurotransmitter receptors

Gene symbol	Receptor (subunit) name
**Norepinephrine**	
ADRA1A	Alpha-1A adrenergic receptor
ADRA1B	Alpha-1B adrenergic receptor
ADRA1D	Alpha-1D adrenergic receptor
ADRA2A	Alpha-2A adrenergic receptor
ADRA2C	Alpha-2C adrenergic receptor
**Acetylcholine**	
CHRM1	Muscarinic Acetylcholine Receptor M1
CHRM2	Muscarinic Acetylcholine Receptor M2
CHRM3	Muscarinic Acetylcholine Receptor M3
CHRM4	Muscarinic Acetylcholine Receptor M4
CHRM5	Muscarinic Acetylcholine Receptor M5
CHRNA2	Nicotinic Cholinergic Receptor (Alpha 2)
CHRNA3	Nicotinic Cholinergic Receptor (Alpha 3)
CHRNA4	Nicotinic Cholinergic Receptor (Alpha 4)
CHRNA6	Nicotinic Cholinergic Receptor (Alpha 6)
CHRNA7	Nicotinic Cholinergic Receptor (Alpha 7)
CHRNA10	Nicotinic Cholinergic Receptor (Alpha 10)
CHRNB1	Nicotinic Cholinergic Receptor (Beta 1)
CHRNB2	Nicotinic Cholinergic Receptor (Beta 2)
**Dopamine**	
DRD1	Dopamine Receptor D1
DRD2	Dopamine Receptor D2
DRD4	Dopamine Receptor D4
**Serotonin**	
HTR1A	5-Hydroxytryptamine Receptor 1A, G protein-coupled
HTR1E	5-Hydroxytryptamine Receptor 1E, G protein-coupled
HTR1F	5-Hydroxytryptamine Receptor 1F, G protein-coupled
HTR2A	5-Hydroxytryptamine Receptor 2A, G protein-coupled
HTR2C	5-Hydroxytryptamine Receptor 2C, G protein-coupled
HTR3B	5-Hydroxytryptamine Receptor 3B, ionotropic
HTR3C	5-Hydroxytryptamine Receptor 3C, ionotropic
HTR4	5-Hydroxytryptamine Receptor 4, G protein-coupled
HTR5A	5-Hydroxytryptamine Receptor 5A, G protein-coupled
HTR7	5-Hydroxytryptamine Receptor 7, G protein-coupled

ADRA2B, CHRNA1, CHRNA5, CHRNA9, CHRNB3, CHRND, CHRNE, DRD3, DRD5, HTR1B, HTR1D, HTR3D, HTR3E, HTR5BP, HTR6 were not included in the analysis as they failed preprocessing steps.

Supplementary Figure 3

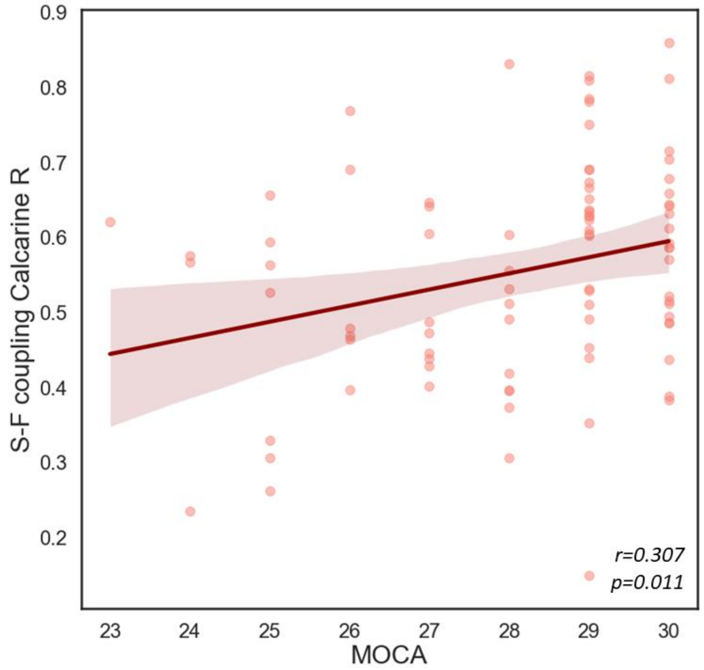

Relationship between SC-FC coupling in the right calcarine gyrus and MOCA scores in patients with Parkinson's Disease (PD).

The p-value shown is corrected for multiple comparisons (comparison of MOCA scores to 3 regions of interest: right calcarine, left insula and right insula).

SC-FC: structural connectivity- functional connectivity

MOCA: Montreal cognitive assessment

Supplementary Figure 4

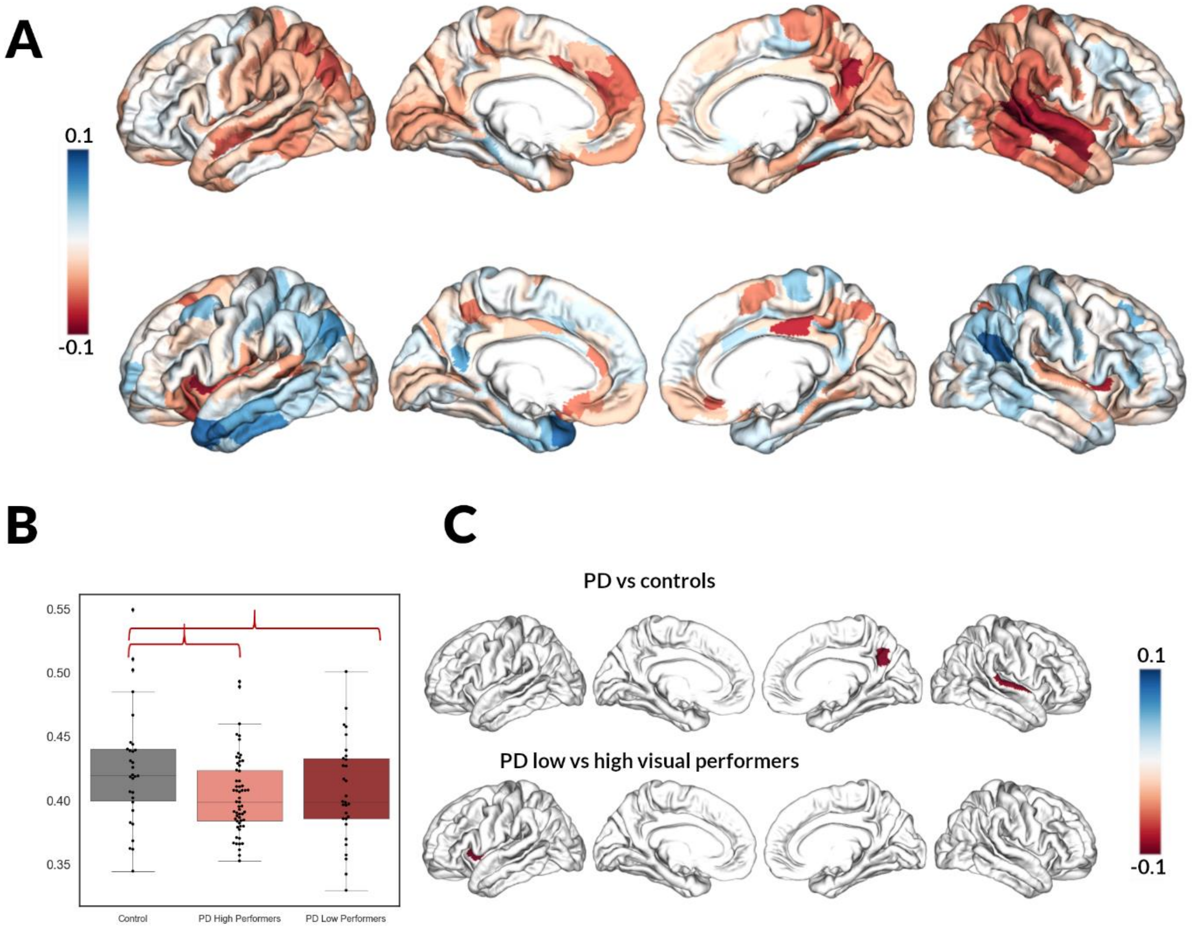

Structure-Function coupling changes in patients with Parkinson's Disease (PD).

Replication using the Glasser Parcellation.

- **A)** Spatial pattern of structure-function decoupling in PD.
  Regional changes in structure-function coupling (correlation coefficient plotted, with age and gender correction) are presented in PD versus controls (top) and PD low visual performers versus PD high visual performers (bottom).
- **B)** Structure-function coupling changes at network level.
  Average structure-function coupling (Spearman's rank correlation) across the whole brain network (400 nodes) is compared between controls, PD high and PD low visual performers. S-F: structure-function. * denotes statistically significant results (p-spin<0.05)
- **C)** Structure-function coupling changes at node level.
  Whole-brain comparisons of structure-function coupling were performed at the node level between PD versus controls (top) and PD low visual performers versus PD high visual performers (bottom), age and gender included as covariates. Only nodes surviving FDR correction (q<0.05) are presented.

Supplementary Figure 5

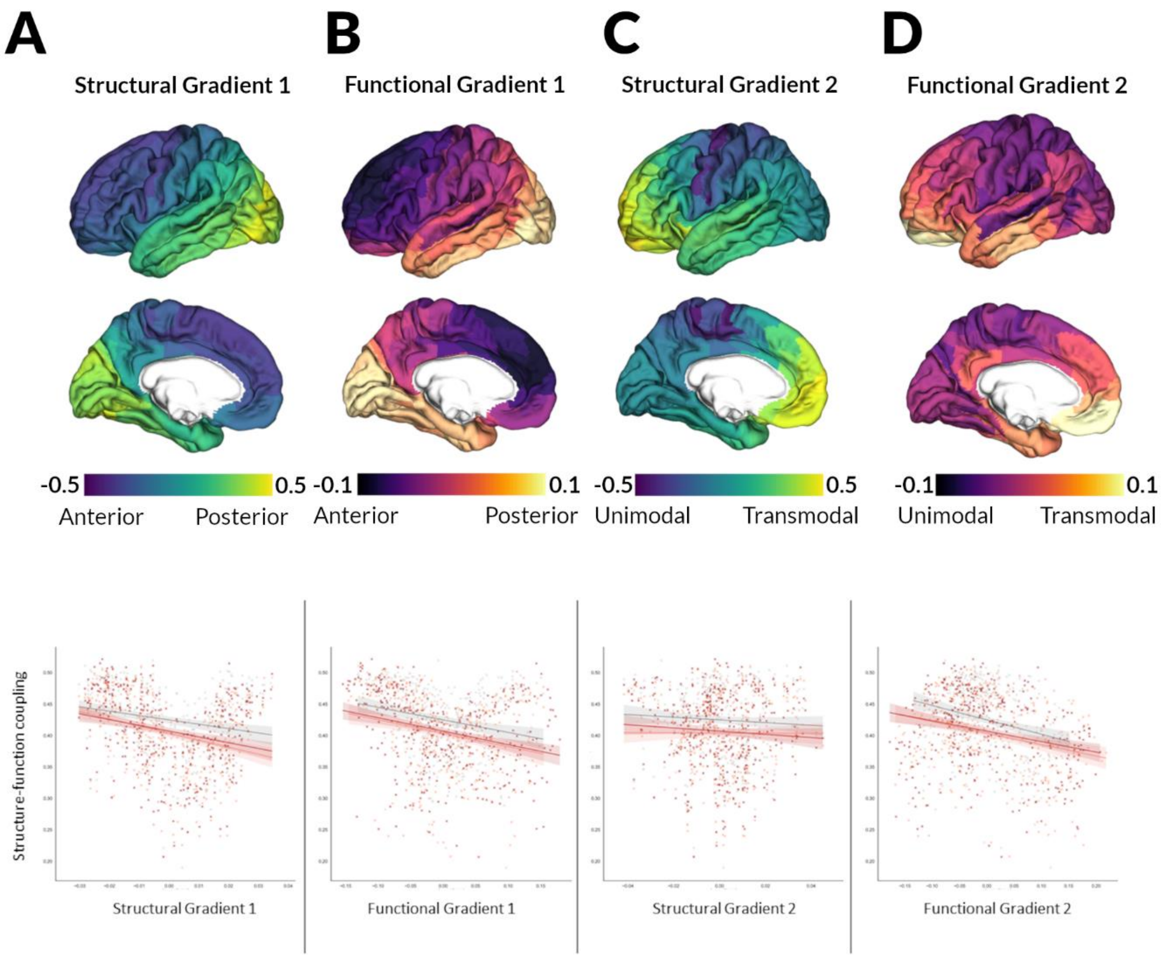

Structure-function decoupling in PD follows macroscale cortical gradients. Replication using the Glasser atlas.

Structure-function coupling is significantly associated with the first principal structural **(A)** and functional gradients **(B)** which align with the anterior-posterior axis. This correlation was seen in all groups but was more pronounced in PD than control participants and even more so in PD low visual performers (who are at higher risk of dementia).

Structure-function coupling also reflected a brain region's position along the second principal structural **(C)** and functional gradients **(D)** which reflect a unimodal-to-transmodal axis. Again this relationship was more pronounced in PD low visual performers then PD high visual performers followed by control participants.

Supplementary Figure 6

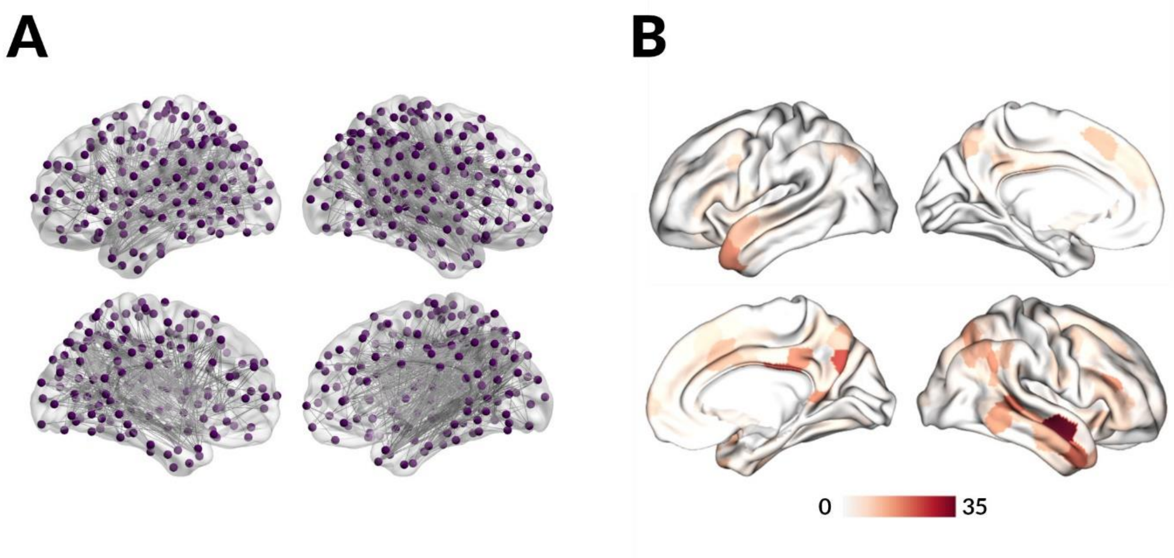

Subnetwork of reduced functional connectivity strength in PD compared to controls.

A. Subnetwork of reduced functional connectivity strength in PD compared to controls (p=0.003). The subnetwork was visualised using BrainNetViewer[2].

B. The nodes of the subnetwork were visualised according to the number of reduced connections; darker red colours represent higher number or connections from that node showing reduced functional connectivity strength (see colour scale).

We performed a network-based statistic (NBS) analysis to investigate whether PD was associated with altered connectivity strength (separately for structural and functional connectivity) in a subnetwork of the brain[3]. A general linear model was used with contrast of interest including PD versus controls and PD low visual performers versus high visual performers with age and gender included as covariates. Permutation testing with unpaired t-tests was performed with 5000 permutations, calculating a test statistic for each connection. A threshold of t = 3.1 as well as family-wise error rate (FWE) of $p<0.05$ was applied.

We found a subnetwork of reduced functional connectivity strength in PD compared to controls (Supplementary Figure S6.A), similar to previously described[4]. No significant subnetwork of reduced structural connectivity strength was seen in PD compared to controls. In addition, no significant subnetworks of reduced structural or functional connectivity strength was seen in PD low visual performers compared to PD high visual performers.

Supplementary Table 3

Supplementary Table 3. Full Transmitter Gene Expression Results

	PD vs controls			PD low visual performers vs PD high visual performers		
**Gene**	**rho**	**p-value**	**q-value**	**rho**	**p-value**	**q-value**
**ADRA1A**	-0.09072	0.071709	0.189	-0.09805	0.051509	0.177
**ADRA1B**	0.077064	0.126253	0.261	-0.05379	0.286234	0.499
**ADRA1D**	-0.08951	0.07558	0.189	-0.03396	0.500894	0.624
**ADRA2A**	0.059949	0.234533	0.330	**-0.13333**	**0.007969**	**0.041**
**ADRA2C**	0.004404	0.930472	0.939	0.053393	0.289798	0.499
**CHRM1**	-0.11423	0.023177	0.090	-0.00061	0.990425	0.990
**CHRM2**	0.031479	0.532757	0.652	-0.0322	0.523375	0.624
**CHRM3**	-0.10441	0.038063	0.131	0.099027	0.049216	0.177
**CHRM4**	0.120468	0.016602	0.074	-0.05725	0.256353	0.497
**CHRM5**	-0.00387	0.938948	0.939	-0.00134	0.978897	0.990
**CHRNA2**	-0.06475	0.199099	0.309	**0.211216**	**2.31E-05**	**0.000**
**CHRNA3**	-0.07305	0.147277	0.285	**-0.19398**	**0.000105**	**0.001**
**CHRNA4**	**0.210163**	**2.54E-05**	**0.000**	**0.180082**	**0.000322**	**0.002**
**CHRNA6**	-0.01043	0.836252	0.926	0.027786	0.581919	0.668
**CHRNA7**	0.005769	0.909012	0.939	0.074356	0.140173	0.334
**CHRNA10**	-0.06118	0.225079	0.330	-0.06766	0.179628	0.398
**CHRNB1**	0.067879	0.17819	0.297	0.03653	0.469096	0.624
**CHRNB2**	-0.06732	0.181827	0.297	-0.08605	0.087632	0.247
**DRD1**	-0.03669	0.46713	0.603	-0.04231	0.401672	0.612
**DRD2**	**-0.16459**	**0.001026**	**0.006**	0.008847	0.86086	0.920
**DRD4**	0.057531	0.253985	0.342	0.033064	0.512324	0.624
**HTR1A**	-0.08308	0.099206	0.220	-0.03947	0.434096	0.612
**HTR1E**	**0.140015**	**0.005309**	**0.027**	**0.236941**	**1.92E-06**	**0.000**
**HTR1F**	**-0.03041**	**0.54672**	**0.652**	0.079703	0.113751	0.294
**HTR2A**	**-0.23976**	**1.43E-06**	**0.000**	0.049985	0.321737	0.525
**HTR2C**	**-0.25124**	**4.22E-07**	**0.000**	-0.08682	0.084827	0.247
**HTR3B**	-0.08839	0.079319	0.189	0.104698	0.037529	0.166
**HTR3C**	0.017708	0.725695	0.833	-0.04108	0.415528	0.612
**HTR4**	**-0.23443**	**2.47E-06**	**0.000**	0.060382	0.231164	0.478
**HTR5A**	0.067976	0.177571	0.297	**0.307555**	**4.23E-10**	**0.000**
**HTR7**	0.094064	0.061802	0.189	0.014368	0.775904	0.859

q-value= FDR-corrected p-value.
With bold results that are significantly different between groups after FDR-correction for multiple comparisons.

URLs

abagen: https://zenodo.org/record/3726257#.XqmYWaj0lPY

Brain Space: https://github.com/MICA-MNI/BrainSpace

BrainSpy (MNI coordinates check): https://github.com/ezPsycho/brainSpy-cli

Schaefer brain parcellation: https://github.com/ThomasYeoLab/CBIG/tree/master/stable_projects/brain_parcellation/Schaefer2018_LocalGlobal

Spatial permutations at parcellation level: https://github.com/frantisekvasa/rotate_parcellation

Code used in the analyses described in this paper: https://github.com/AngelikaZa/SCFC

References:

1. Weil, R. S. *et al.* The Cats-and-Dogs test: A tool to identify visuoperceptual deficits in Parkinson's disease. *Mov. Disord.* **32**, 1789–1790 (2017).
2. Xia, M., Wang, J. & He, Y. BrainNet Viewer: A Network Visualization Tool for Human Brain Connectomics. *PLOS ONE* **8**, e68910 (2013).
3. Zalesky, A., Fornito, A. & Bullmore, E. T. Network-based statistic: Identifying differences in brain networks. *NeuroImage* **53**, 1197–1207 (2010).
4. Abós, A. *et al.* Discriminating cognitive status in Parkinson's disease through functional connectomics and machine learning. *Sci. Rep.* **7**, 45347 (2017).
