# Supplementary material for: Organisational and neuromodulatory underpinnings of structural-functional connectivity decoupling in patients with Parkinson’s disease
Source: Commun Biol. 2021 Jan 19;4:86. doi: 10.1038/s42003-020-01622-9 (PMC7815846; doi:10.1038/s42003-020-01622-9)
Supplement: Supplementary file 3 — Description of Additional Supplementary Files [file 42003_2020_1622_MOESM3_ESM.pdf]

Description of Additional Supplementary Files

File Name: Supplementary Dataset
Description:

Supplementary Data 1 Zip File

1. File Name: Fig1_AverageFuncConnectome_Control.csv
   Description: Figure 1 Source Data
2. File Name: Fig1_ExampleFuncConnectome_400_norm_bin.csv
   Description: Figure 1 Source Data
3. File Name: Fig1_ExampleStructureConnectome_norm_400.csv
   Description: Figure 1 Source Data
4. File Name: Fig1_FunctionalGradients.csv
   Description: Figure 1 Source Data
5. File Name: Fig1_StructuralGradients.csv
   Description: Figure 1 Source Data
6. File Name: Figure1.ipynb
   Description: Figure 1 Code as Jupyter Notebook

Supplementary Data 2 Zip File

1. File Name: Fig2_ClinicalInformation.xlsx
   Description: Figure 2 Source Data
2. File Name: Fig2_StructureFunction.csv
   Description: Figure 2 Source Data
3. File Name: Fig2_StructureFunction_PD.csv
   Description: Figure 2 Source Data
4. File Name: Fig2_StructureFunction_PD_VisPerf.csv
   Description: Figure 2 Source Data
5. File Name: Figure2.ipynb
   Description: Figure 2 Code as Jupyter Notebook

Supplementary Data 3 Zip File

1. File Name: Fig3_Axes.csv
   Description: Figure 3 Source Data
2. File Name: Fig3_GradientsControls.csv
   Description: Figure 3 Source Data
3. File Name: Figure3.ipynb
   Description: Figure 3 Code as Jupyter Notebook

Supplementary Data 4 Zip File

1. File Name: Fig4_FunctionalGradientOne_NA.csv
   Description: Figure 4 Source Data
2. File Name: Fig4_FunctionalGradientTwo_NA.csv
   Description: Figure 4 Source Data
3. File Name: Fig4_StructuralGradientOne_NA.csv
   Description: Figure 4 Source Data
4. File Name: Fig4_StructuralGradientTwo_NA.csv

Description: Figure 4 Source Data

5. File Name: Figure4.ipynb
   Description: Figure 4 Code as Jupyter Notebook

Supplementary Data 5 Zip File

1. File Name: Fig5_GeneCorrelation_PD.csv
   Description: Figure 5 Source Data
2. File Name: Fig5_GeneCorrelation_PDVisperf.csv
   Description: Figure 5 Source Data
3. File Name: Figure5.ipynb
   Description: Figure 5 Code as Jupyter Notebook
