# Supplementary material for: Organisational and neuromodulatory underpinnings of structural-functional connectivity decoupling in patients with Parkinson’s disease
Source: Commun Biol. 2021 Jan 19;4:86. doi: 10.1038/s42003-020-01622-9 (PMC7815846; doi:10.1038/s42003-020-01622-9)
Supplement: Supplementary file 5 — Reporting Summary [file 42003_2020_1622_MOESM5_ESM.pdf]

nature research

Corresponding author(s): Angeliki Zarkali

Last updated by author(s): 09/10/2020

Reporting Summary

Nature Research wishes to improve the reproducibility of the work that we publish. This form provides structure for consistency and transparency in reporting. For further information on Nature Research policies, see our Editorial Policies and the Editorial Policy Checklist.

Statistics

For all statistical analyses, confirm that the following items are present in the figure legend, table legend, main text, or Methods section.

n/a	Confirmed	
☐	☒	The exact sample size (*n*) for each experimental group/condition, given as a discrete number and unit of measurement
☐	☒	A statement on whether measurements were taken from distinct samples or whether the same sample was measured repeatedly
☐	☒	The statistical test(s) used AND whether they are one- or two-sided<br>*Only common tests should be described solely by name; describe more complex techniques in the Methods section.*
☐	☒	A description of all covariates tested
☐	☒	A description of any assumptions or corrections, such as tests of normality and adjustment for multiple comparisons
☐	☒	A full description of the statistical parameters including central tendency (e.g. means) or other basic estimates (e.g. regression coefficient) AND variation (e.g. standard deviation) or associated estimates of uncertainty (e.g. confidence intervals)
☐	☒	For null hypothesis testing, the test statistic (e.g. F, t, r) with confidence intervals, effect sizes, degrees of freedom and P value noted<br>*Give P values as exact values whenever suitable.*
☒	☐	For Bayesian analysis, information on the choice of priors and Markov chain Monte Carlo settings
☒	☐	For hierarchical and complex designs, identification of the appropriate level for tests and full reporting of outcomes
☐	☒	Estimates of effect sizes (e.g. Cohen's *d*, Pearson's *r*), indicating how they were calculated

Our web collection on statistics for biologists contains articles on many of the points above.

Software and code

Policy information about availability of computer code

Data collection	Demographic, clinical and psychometric data was collected and stored in excel. MRI data was collected using a 3T Siemens scanner and processed using Mrtrix 3.0 for diffusion data and Nipype for resting state fmri data. All steps of data processing are described in the manuscript.
Data analysis	Gradients were derived using BrainSpace v1.0. Statistical analyses were performed in Python 3 using Jupyter Lab v.1.0.2. All analyses are described in full in the manuscript.

For manuscripts utilizing custom algorithms or software that are central to the research but not yet described in published literature, software must be made available to editors and reviewers. We strongly encourage code deposition in a community repository (e.g. GitHub). See the Nature Research guidelines for submitting code & software for further information.

Data

Policy information about availability of data

All manuscripts must include a data availability statement. This statement should provide the following information, where applicable:
- Accession codes, unique identifiers, or web links for publicly available datasets
- A list of figures that have associated raw data
- A description of any restrictions on data availability

All data and results of statistical analyses are presented in the manuscript. Code will be made available on github (https://github.com/AngelikaZa?tab=repositories) prior to publication. Individual level data will be made available to researchers upon request.

Field-specific reporting

Please select the one below that is the best fit for your research. If you are not sure, read the appropriate sections before making your selection.

☒ Life sciences ☐ Behavioural & social sciences ☐ Ecological, evolutionary & environmental sciences

For a reference copy of the document with all sections, see nature.com/documents/nr-reporting-summary-flat.pdf

Life sciences study design

All studies must disclose on these points even when the disclosure is negative.

Sample size	Participants were recruited to the Vision in Parkinson's disease study, a longitudinal observation study. The study was powered to be able to detect differences in high level visual performance at baseline for participants with Parkinson's disease.
Data exclusions	From our initially recruited cohort 4 participants were excluded for failing quality control criteria for diffusion weighted imaging and 11 participants for failing quality control criteria for resting state fmri imaging. This lead to 118 participants were included in our study (88 patients with PD and 30 controls), for which results are presented in the study.
Replication	The findings of this study can not be replicated in another cohort, as existing publicly available cohorts of patients with Parkinson's disease do have not collected equivalent visuoperceptual tasks. To ensure robustness of our results however we have replicated our analysis using a different brain parcellation.
Randomization	This was an observational study therefore no randomisation took place.
Blinding	he researchers collecting clinical and imaging data were blinded during data collection. Groups were derived later from participant performance on visual tasks and not allocated by the researchers. T

Reporting for specific materials, systems and methods

We require information from authors about some types of materials, experimental systems and methods used in many studies. Here, indicate whether each material, system or method listed is relevant to your study. If you are not sure if a list item applies to your research, read the appropriate section before selecting a response.

Materials & experimental systems

n/a	Involved in the study	
☒	☐	Antibodies
☒	☐	Eukaryotic cell lines
☒	☐	Palaeontology and archaeology
☒	☐	Animals and other organisms
☐	☒	Human research participants
☐	☒	Clinical data
☒	☐	Dual use research of concern

Methods

n/a	Involved in the study	
☒	☐	ChIP-seq
☒	☐	Flow cytometry
☐	☒	MRI-based neuroimaging

Human research participants

Policy information about studies involving human research participants

Population characteristics	A total of 118 participants were included: 88 patients with PD and 30 controls. Patients with PD were further classified according to their performance in two higher-order computer-based visual tasks resulting in 33 PD low visual performers and 55 PD high visual performers. Mean age was 66.2 years, 62.7% male (74 participants).
Recruitment	We included 88 patients with PD, recruited to our London centre from clinics in the National Hospital for Neurology and Neurosurgery and affiliated hospitals. All patients with PD fullfilled the Queen Square Brain Bank Criteria. We also recruited 30 unaffected controls from volunteer databases in our institution and patient spouses.
Ethics oversight	Queen Square Ethics committee. REC reference: 15/LO/0476.

Note that full information on the approval of the study protocol must also be provided in the manuscript.

Clinical data

Policy information about clinical studies

All manuscripts should comply with the ICMJE guidelines for publication of clinical research and a completed CONSORT checklist must be included with all submissions.

Clinical trial registration	REC reference: 15/LO/0476
Study protocol	The study protocol and other information about the study can be found here: https://vision-in-parkinsons.co.uk/
Data collection	All clinical and imaging data was collected over a single study visit. Clinical and psychological assessments were performed at the Institute of Neurology, Queen Square, University College London. All imaging data was collected at the same scanner (3T Siemens) at the Wellcome Centre for Neuroimaging, University College London.
Outcomes	This was an observational study. Participants with PD were classified according to their performance in two computer-based higher-order visual tasks (Cats and Dogs task, and Biological motion task). Participants with PD were classified as low visual performers (n=30), if they performed worse than the group median on both tasks. All other patients with PD were classified as high visual performers (n=58).

Magnetic resonance imaging

Experimental design

Design type	Resting state.
Design specifications	N/A
Behavioral performance measures	N/A

Acquisition

Imaging type(s)	Resting state functional MRI.
Field strength	3T
Sequence & imaging parameters	All MRI data were acquired on a 3T Siemens Magnetom Prisma scanner (Siemens) with a 64-channel head coil. Resting state functional MRI (rsfMRI) was acquired with the following parameters: gradient-echo EPI, TR=70ms, TE=30ms, flip angle=90°, FOV=192×192, voxel size=3x3x2.5 mm, 105 volumes, 7-minute session. During rsfMRI, participants were instructed to lie quietly with their eyes closed and avoid falling asleep; this was confirmed by monitoring and post-scan debriefing. A 3D MPRAGE (magnetization prepared rapid acquisition gradient echo) image (voxel size=1×1×1 mm, TE=3.34ms, TR= 2530 ms, flip angle=7°) was also obtained. Imaging for all participants was performed at the same time of day, with PD participants receiving their normal medications.
Area of acquisition	Whole brain scan

Diffusion MRI ☒ Used ☐ Not used

Parameters: Diffusion weighted imaging (DWI) was acquired with the following parameters: b0 in both AP and PA directions, b=50 s/mm2 /17 directions, b=300 s/mm2 /8 directions, b=1000 s/mm2 /64 directions, b=2000 s/mm2 /64 directions, 2x2x2 mm isotropic voxels, TE=3260ms, TR=58ms, 72 slices, 2mm thickness, acceleration factor = 2. DWI acquisition time was approximately 10 min.

Preprocessing

Preprocessing software	Pre-processing of DWI images was performed in MRtrix3.0.<br>rsfMRI data underwent standard pre-processing using fMRIPrep 1.5.0.
Normalization	Diffusion weighted imaging were analysed at individual space. he raw T1-weighted images were registered to the diffusion-weighted image using NiftyReg and five-tissue anatomical segmentation performed using the 5ttgen script in MRtrix. Resting state fmri images were normalised to MNI space. Motion correcting transformations, field distortion correcting warp, BOLD-to-T1w transformation and T1w-to-template (MNI) warp were concatenated and applied in a single step using antsApplyTransforms (ANTs v2.1.0) using Lanczos interpolation.
Normalization template	MNI 2009 for resting state fMRI data.
Noise and artifact removal	Diffusion weighted images underwent denoising, removal of Gibbs artefacts, eddy-current and motion correction and bias field correction. Diffusion tensor metrics were calculated and constrained spherical deconvolution performed.<br>Functional data was slice-time corrected using 3dTshift from AFNI106 and motion corrected using mcflirt. Distortion correction was performed using a TOPUP implementation. This was followed by co-registration to the corresponding T1-weighted image using boundary-based registration with six degrees of freedom.<br>Physiological noise regressors were extracted applying CompCor. Sources of spurious variance were removed through linear regression (six motion parameters, mean signal from white matter and cerebrospinal fluid), followed by calculation of bivariate correlations and application of Fisher transform.

Volume censoring	For resting state fMRI, the first 4 volumes were discarded to allow for steady state equilibrium.

Statistical modeling & inference

Model type and settings	For group comparisons between structure-function coupling and gradient component scores we used general linear model, with age and gender as covariates and comparisons of interest: 1) PD versus controls and 2) PD low visual performers versus PD high visual performers.
Effect(s) tested	We tested regional differences between structural and functional connectivity strength coupling between 1) PD versus controls and 2) PD low versus high visual performers.<br>We correlated these regional differences in coupling to gradient component scores and neurotransmitter receptor genes using Spearman correlation coefficient

Specify type of analysis: ☐ Whole brain ☒ ROI-based ☐ Both

Anatomical location(s)	400 cortical regions of interest (ROIs) were generated by segmenting each participant's T1-weighted image using the Schaefer parcellation. We replicated structure-function analyses using the Glasser parcellation.
Statistic type for inference (See Eklund et al. 2016)	General linear model.
Correction	We controlled for multiple comparisons using the False Discovery Rate (Benjamini-Hochberg method, q<0.05).

Models & analysis

n/a	Involved in the study
☐	☒ Functional and/or effective connectivity
☒	☐ Graph analysis
☒	☐ Multivariate modeling or predictive analysis

Functional and/or effective connectivity	Functional connectivity between ROIs was quantified as the Pearson correlation coefficient between mean regional BOLD time series.
